# Quickly SOFA Score Can Be Used as a High-Efficiency Classified Method for COVID-19 Infected Patients

**DOI:** 10.18502/ijph.v49i8.3915

**Published:** 2020-08

**Authors:** Ke FENG, Zhongwei CHEN, Bin MEI, Xing Du, Xufeng FU

**Affiliations:** 1. Department of Emergency, General Hospital of Ningxia Medical University, Yinchuan, Ningxia, China; 2. Department of Neurology, Wuhan University Zhongnan Hospital, Wuhan, Hubei, China; 3. Key Laboratory of Fertility Preservation and Maintenance of Ministry of Education, Ningxia Medical University, Yinchuan, Ningxia, China

## Dear Editor-in-Chief

The outbreak of the COVID-19 caused a substantial public health crisis in Wuhan, China, and then expeditiously spread all over China. CoVID-19 is a “public-health emergency of international concern” due to the pandemic is escalating rapidly (1).

At the beginning of February, 2020, thousands of people are infected every day, then the Chinese government rapidly built two hospitals (Huoshenshan Hospital and Leishenshan Hospital) at an alarming rate for severe patients’ treatment, and swiftly converted 16 large-scale public places in Wuhan into Fangcang shelter hospitals for patients with the COVID-19 of mild symptoms (2). The Fangcang shelter hospitals isolated COVID-19 positive patients and provided high-quality medical treatment and care, as well as played an important role in triage function to classify based on the severity of the disease. In the these hospitals, the patients who deteriorate from mild to severe urgently need to be transferred to the high-level or specialized respiratory hospital for treatment. Therefore, it was critical to rapidly identify individuals who became severe or even critically ill upon infection, for the purposes of ensure the safety of mild patients, medical staff and hospital staff, as well as the most effective use of medical and monitoring equipment, especially in the absence of specific drugs directly targeting at COVID-19.

In this study, we explored the 437 cases and transferred 33 becoming critically ill cases to higher-level hospitals by using vital sign analysis and quickly Sequential Organ Failure Assessment (qSOFA) score system. We found the results of vital signs analysis and qSOFA score were basically consistent with the evaluation criteria of transferred to high-level hospitals. Therefore, qSOFA score would likely be a preliminary and rapid method for identification and classification that can maximize the safety of hospital workers and make effective use of medical equipment.

In order to maximize the function of the Fangcang shelter hospitals, we made the following suggestions:

1. The patients would be scored according to the criteria of qSOFA score after entering the hospital, and the score was performed every three days. The patients got 1 point when they showed one of the following symptoms respectively: systolic pressure ≤100 mmHg; respiratory rate ≥22 times/min; mental state <13pionts (Table 1).
2. If the patient showed qSOFA score=1 point, he/she should be transferred to public ward of high-level or special hospital for closely monitoring
3. If the qSOFA score was ≥2 points, he/she must be transferred to the high-level or special hospital and monitored in the intensive care unit (ICU) for further treatment immediately. In addition, it was required a re-evaluation of the qSOFA score after entering the ICU.

Overall, 437 COVID-19 patients were included for analysis, with 33 (7.6%) cases became severe and transferred to high-level hospital. The sever cases transferred to high-level hospital were elderly, lower level in oxyhemoglobin saturation and body temperature, higher level in respiratory rate, more obvious fever (Table 2).

**Table 1:** The criteria of Glasgow Coma Score (3)

***Response***	***Scale***	***Score***
Eye opening response	Eyes open spontaneously	4 points
	Eyes open to verbal command, speech or shout	3 points
	Eyes open to pain (nit applied to face)	2 points
	No eyes open	1 point
Verbal response	Oriented	5 points
	Confused conversation, but able to answer questions	4 points
	Inappropriate responses, words discernible	3 points
	Incomprehensible sounds or speech	2 points
	No verbal response	1 point
Motor response	Obeys commands for movement	6 points
	Purposeful movement to painful stimulus	5 points
	Withdraws from pain	4 points
	Abnormal (spastic) flexion	3 points
	Extensor (rigid) response	2 points
	No motor response	1 point

**Table 2:** Demographic information dependent on qSOFA score of patients with confirmed COVID-19 infection in Fangcang Shelter Hospital

*****Variables*****	*****Total (N= 437)*****	*****None-transfer to high-level hospital (N=404)*****	*****Transfer to high-level hospital (N=33)*****	*****P-value*****
Age(yr)	54(42, 61)	54 (41, 60.75)	61 (51, 62)	0.004
Sex				0.170
Male	228 (52.2%)	207 (51.2%)	21 (63.6%)	
Female	209 (47.8%)	197 (48.8%)	12 (36.4%)	
Vital signs				
HR	82 (78, 88)	82(78, 88)	80 (75.5, 90)	0.605
SaO2	96 (94, 98)	96 (95, 98)	95 (92.5, 96)	<0.01
T	36.6 (36.5, 36.8)	36.7 (36.5, 36.8)	36.5 (36.4, 36.6)	0.002
RR	18 (16, 20)	18 (15, 20)	21 (19, 23)	<0.01
Fever				0.003
Yes	248 (56.8%)	238 (58.9%)	10 (30.3%)	
No	189 (43.2%)	166 (41.1%)	23 (69.7%)	
Cough				0.172
Yes	78 (17.8%)	75 (18.6%)	3 (9.1%)	
No	359 (82.2%)	329 (81.4%)	30 (90.9%)	
qSOFA score				<0.01
0 point	404 (92.5%)	404 (100%)	0	
1 point	25 (5.7%)	0	25 (75.8%)	
≥2 points	8 (1.8%)	0	8 (24.2%)	

Data are expressed as mean±standard deviation (SD), median (interquartile range), or number (percent). Comparisons between none-transfer and transfer to high-level hospital cases were performed by a chi-square test. HR: heart rate (beat/min); SaO2: oxyhemoglobin saturation (%); T: body temperature (°C); RR: respiratory rate (times/min).

In addition, 25 cases scored 1 point and 8 cases scored ≥2 points after qSOFA score. No death was reported by the end of follow-up.

These results showed a similar trend between clinical features (including SaO2, T, RR and fever) and qSOFA score, which could instruct doctors to transfer sever COVID-19 infected patients to high-level hospital as soon as possible. Therefore, the qSOFA score could be regard as a rapid identification method to identify the patients whose condition deteriorate. It is helpful for designing specific strategies for prevention and treatment of this disease.

## Funding

This work was supported by the Key Research and Development Program of Ningxia (No. 2019BEG03043) and the West China first-class

Disciplines Basic Medical Sciences at Ningxia Medical University (No. NXYLXK2017B07).
